# Circadian melatonin profile in opium and amphetamine dependent patients: A preliminary study

**DOI:** 10.1016/j.nbscr.2019.100046

**Published:** 2019-08-01

**Authors:** Habibolah Khazaie, Hamid Reza Ahmadi, Amir Kiani, Mohammad Rasoul Ghadami

**Affiliations:** a Sleep Disorders Research Center, Kermanshah University of Medical Sciences, Kermanshah, Iran; b Department of Pharmacology and Toxicology, School of Pharmacy, Kermanshah University of Medical Sciences, Kermanshah, Iran

**Keywords:** Amphetamine, Opium, Melatonin, Amphetamine, Opium, Melatonin, Sleep disturbance, Circadian rhythm

## Abstract

**Aim:**

The aim of this study was to investigate the relationship between opium and amphetamine dependency with the serum melatonin levels in the presence of circadian rhythm sleep disorders (CRSD).

**Participants:**

Forty four male amphetamine-dependent and opium-dependent patients with CRSD and with more than one year substance dependency were enrolled in this study. Control group consisted of twelve healthy male subjects.

**Design:**

The diagnoses of sleep disorders were established by a psychiatrist and were made on the basis of the criteria of ICSD-II using the patients’ sleep logs. Blood samples were drawn every 4 h through an intravenous catheter. Serum melatonin levels were assayed using an enzyme-linked immunosorbent assay (ELISA) kit. Repeated Measures Analysis of variance (ANOVA) was used to assess differences between the melatonin levels at six separate times.

**Finding:**

The serum melatonin levels of the control subjects were significantly higher than both opium-dependent and amphetamine-dependent patients at 24:00, 4:00 and 8:00. The serum melatonin level of the opium-dependent patients were significantly lower than the amphetamine-dependent patients at 24:00 (26.9 ± 11.4 vs. 41 ± 19.4, respectively; p = 0.006) and were significantly higher than the amphetamine-dependent patients at 16:00 (12.7 ± 5.1 vs. 8.9 ± 4.1, respectively; p = 0.011).

**Conclusion:**

This is an evidence of negative effects of substance dependence on circadian cycle of melatonin secretion among opium and amphetamine dependent patients.

## Highlights

• Sleep problems in substance abuse patients may be caused by dysfunction of circadian rhythm.
• High prevalence of circadian rhythm sleep disorders (CRSD) in substance dependent patients, possibly related to abnormal melatonin cycle.
• Circadian rhythm-based interventions could play an important role in the prevention and treatment of substance use disorders.

## 1 Introduction

Amphetamine derivatives produce long-term neurotoxic effects on the central nervous system (Permpoonputtana et al., 2012). During the past decades, the amphetamines derivatives abuse become an important public health problem.

Although acute administration of low doses of methamphetamine has positive subjective effects on mood and cognitive performance (Hart et al., 2002; Johnson et al., 2000), long-term abuse of larger doses has deleterious effects and is associated with cognitive impairments and mood disturbances (London et al., 2004, 2005).

Most drugs can adversely affect sleep patterns and have negative impact on both the duration and frequency of sleep stages (Barkoukis and Avidan, 2007). Using any sedative and narcotic substance makes some changes in sleep; moreover, quitting and withdrawing of this substance makes cause sleep disturbances (KaplanSadock, 2009). The relationship between substance abuse and sleep is bidirectional in that substance use may directly lead to sleep disturbances, and sleep disturbances may be a risk factor for relapse to substance use (Kurth et al., 2009; Hasler et al., 2012).

There is no study described the characteristics of sleep disorders in substance abuse patients in accordance with the criteria of the 2nd edition of the International Classification of Sleep Disorders (ICSD-II). On the other hand, previous studies showed that one of the main causes of the chronic sleep problems is circadian rhythm sleep disorders (CRSD) that affected by biological, behavioral and environmental factors (Zhu and Zee, 2012; Wagner, 1999). Melatonin, a major hormone of pineal gland, has a marked circadian variation that is controlled by the central circadian pacemaker in the suprachiasmatic nuclei of the hypothalamus (Claustrat et al., 2005). Although it seems that sleep problems in substance abuse patients may be caused by dysfunction of circadian rhythm, there is no study that examined the relationship between the serum melatonin levels and the presence of CRSD in substance dependents. Our hypothesis in this study was that serum melatonin levels are different in opium and amphetamine dependent patients in the presence of CRSD.

### 1.1 Subjects and methods

This study was approved by the ethics committee of Kermanshah University of Medical Sciences, Kermanshah, Iran. Written informed consent was obtained from the all participants. Forty four male amphetamine-dependent and opium-dependent patients were recruited for the study from the Farabi Hospital, Kermanshah. We enrolled patients who had received diagnoses of substance dependency by psychiatry interview, based on DSM-IV criteria and currently drug free awaiting treatment for their dependency. The patients filled out their sleep log for at least three weeks and they were in the hospital at the night of sampling and the night before sampling, to keep study condition.

The inclusion criteria for the study were: men between the age's 20 and 55, DSM-IV criteria for amphetamine or opium dependency and more than one year opium and amphetamine dependency. However, substance dosage was not recorded. The exclusion criteria were: dependence on any substance other than amphetamine and opium, any other major psychiatric diagnosis, and taking any medication that can affect serum melatonin levels such as alcohol, benzodiazepines and caffeine. We also excluded patients who were suffering from any physical infirmities. Twelve healthy male subjects without any psychiatric, neurological, addictive or sleep problems consisted as control group.

Although to establish a diagnosis of CRSD, use of objective assessments such as actigraphic recording is more validated, because of our study limitation, we did not record objective variables and the diagnoses of sleep disorders were established by a psychiatrist and were made on the basis of the criteria of ICSD-II by using the patients’ sleep logs and psychiatric interview. Blood samples were drawn every 4 h through an intravenous catheter inserted continuously for 24 h into a forearm vein. A heparin lock was used to prevent clotting. Light intensity of the facilities was kept under 15 lux at nighttime to prevent the suppression of nocturnal melatonin secretion by bright light. However, daytime luminance was not controlled and recorded. The blood samples were centrifuged and serums were separated within 30 min and were incubated at −70 °C. Serum melatonin levels were assayed using an enzyme-linked immunosorbert assay (ELISA) kit (cat no. RE54021; IBL, Hamburg, Germany) with the following characteristics: sensitivity, 1.6 pg/ml; intra-assay coefficient of variation (CV), 3.0–11.4%; inter-assay CV, 4–19.3%) using the standard protocol (Khaleghipour et al., 2012).

#### 1.1.1 Statistical analysis

Statistical analyses were performed using SPSS (version 16.0). The Student's *t*-test, ANOVA and chi square test were used for the comparison of variables between the groups. Repeated Measures Analysis of variance (ANOVA) was used to assess differences between the melatonin levels at six separate times (i.e. every 4 h). Post-hoc Tukey tests were used for comparisons of significant effects identified by ANOVA. The Logistic regression was used for identify correlation between the duration of substance use and melatonin level. A p-value of less than 0.05 was considered to represent a statistically significant difference.

## 2 Results

Forty four patients were identified (total population, 62 patients) with sleep disorders based on the psychiatric interview and ICSD-II criteria. From 33 opium-dependent patients, 22 patients (66%) and from 29 amphetamine-dependent patients, 22 patients (75%) received diagnoses of circadian rhythm sleep disorders (CRSD) based on ICSD-II criteria (p: 0.426). Demographics characteristics of study participants are listed in Table 1. A comparison of the variables between the opium-dependent patients and the amphetamine-dependent patients did not reveal any significant differences between the two groups in CRSD type (In the opium-dependent group, irregular sleep–wake type [ISWT], n = 8; delayed sleep phase type [DSPT], n = 14; and in the amphetamine-dependent group, irregular sleep–wake type [ISWT], n = 5; delayed sleep phase type [DSPT], n = 17; chi-square test, p: 0.322). However, the duration of substance use in opium-dependent patients was significantly longer than amphetamine-dependent patients (t. test, p < 0.0001).

**Table 1:** Patients demographic characteristics.

	Opium dependents (n = 22)	Amphetamine dependents (n = 22)	Control subjects (n = 12)	p value
Age (year)	34.5 ± 10.5	29.9 ± 6.1	31.3 ± 5.8	0.246
Duration of use (year)	10.1 ± 8.2	2.3 ± 1.4	–	<0.0001
ICSD II, n (%) –				
*Initial*	14 (64)	17 (77)		0.322
*Irregular*	8 (36)	5 (23)		
Total sleep duration during nighttime (hour)	7.8 ± 1.5	7.4 ± 1.9	7.2 ± 1.4	0.242
Total sleep duration during daytime (hour)	2.3 ± 0.3	2.1 ± 0.5	1.9 ± 0.5	0.079
Number of Awakening after Sleep Onset (n)	4.1 ± 2.4	4.8 ± 3	0.8 ± 1.1	<0.0001
Sleep latency (min)	41 ± 31	47 ± 26	14 ± 9	<0.0001

Data presented as mean ± SD or number (%).

There were no significant differences in total sleep duration during nighttime (opium-dependent patients, 7.8 ± 1.5 h vs. amphetamine-dependent, 7.4 ± 1.9 h; vs. control subjects, 7.2 ± 1.4 h; p = 0.242) and total sleep duration during daytime (opium-dependent patients, 2.3 ± 0.3 h; amphetamine-dependent, 2.1 ± 0.5; control subjects, 1.9 ± 0.5 h; p = 0.079) between the groups.

However, mean number of awakening after sleep onset were significantly lower in control group than both substance dependent groups (p < 0.0001) and there were no significant differences between opium and amphetamine dependent group (p = 0.572). Also, controls had lower sleep latency than both substance dependents (p < 0.0001). There were no significant differences between opium and amphetamine dependent group regarding to sleep latency (p = 0.429).

**Fig. 1:**
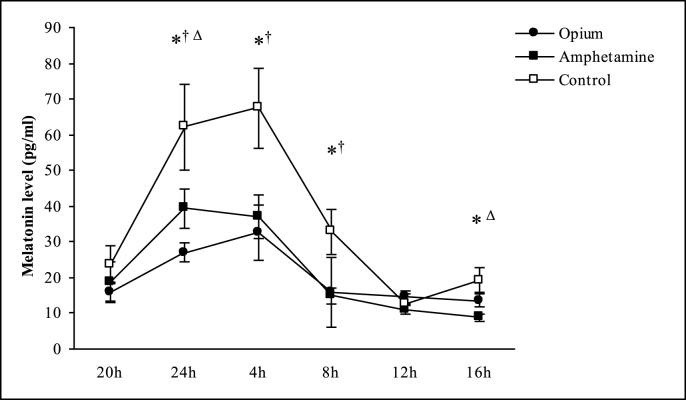
Comparison of the serum melatonin levels at six time measurement points between study groups (significant difference indices (p < 0.05) ^∗^Control vs. Amphetamine, ^†^ Control vs. Opium, ^Δ^ Opium vs. Amphetamine).

To compare the serum melatonin levels between the opium-dependent patients and amphetamine-dependent patients, two-way repeated ANOVA (group × time measurement point) was repeated (Fig. 1). The time measurement point showed a significant effect on serum melatonin level (F[5, 265]) = 40.44, p < 0.0001, and also significant differences in the serum melatonin levels were observed for the groups or a significant interaction between the groups and the time measurement point (main effect of the groups: F[2,53] = 199.57, p < 0.0001; interaction between the groups and the time measurement point: F[5265] = 3.42, p < 0.001). Post-hoc analysis showed that the serum melatonin levels of the control subjects were significantly higher than both opium-dependent and amphetamine-dependent patients at 24:00, 4:00 and 8:00 (p < 0.005). On the other hand the serum melatonin level of the amphetamine-dependent patients (8.9 ± 4.1 pg/ml) were significantly lower than the opium-dependent patients (12.7 ± 5.1 pg/ml) as well as control subjects (17.7 ± 12.3 pg/ml) at 16:00 (p = 0.011 for amphetamine-dependent vs. opium-dependent; p = 0.006 for amphetamine-dependent vs. control). However, there was no significant difference between opium-dependents and controls (p = 0.129).

Furthermore, opium-dependent patients had significantly lower serum melatonin level than the amphetamine-dependent patients at 24:00 (26.9 ± 11.4 pg/ml vs. 41 ± 19.4 pg/ml, respectively; p = 0.006). There were no significant differences in the serum melatonin levels between the three study groups at other time measurement points.

Regarding the relationship between duration of substance dependency and serum melatonin level, there was no significant correlation between these two variables was observed in the both groups (Table 2).

**Table 2:** Correlation between duration of substance dependency and serum melatonin level at six time measurement points.

Time	Opium dependents (n = 22)		Amphetamine dependents (n = 22)	
	*β*	*Sig*	*β*	*Sig*
4 h	0.084	0.812	0.652	0.054
8 h	−0.286	0.355	−0.333	0.258
12 h	0.308	0.307	−0.150	0.567
16 h	−0.487	0.290	0.337	0.185
20 h	0.643	0.143	−0.106	0.652
24 h	−0.432	0.196	−0.114	0.730

## 3 Discussion

To the best of our knowledge, this is the first study in which the serum melatonin level of the opium-dependent patients and amphetamine-dependent patients have been compared. Our results showed that the serum melatonin level of the both opium-dependent and amphetamine-dependent patients were significantly lower than healthy controls. Also, opium-dependent patients were significantly lower than the amphetamine-dependent patients at night and the serum melatonin level of the opium-dependent patients were significantly higher than the amphetamine-dependent patients in the evening. Also, there was no correlation between duration of substance use and melatonin level in both groups.

This is an evidence of the effects of substance dependency on 24-h cycle melatonin. Melatonin secretion changes have not been clearly investigated in substance abuser. In a study by Hasler et al., the relationship between salivary melatonin-based dim light melatonin onsets (DLMOs) and sleep parameters in adolescents with a history of substance abuse and current sleep difficulties was examined. The results of their study showed that when the subjects divided into late and early DLMOs, the two groups had significantly different phase angles between DLMO and sleep variables but no other significant differences in sleep parameters. However, they concluded that circadian phase and its relationship to sleep may have sleep and behavioral consequences (Hasler et al., 2008).

All patients in our study have the sleep-wake cycle disorders (including starting, continuing or irregular cycles). It can be linked with lower levels of melatonin in these patients. Melatonin is a hormone in healthy subjects with normal nighttime sleep at night, at 2 a.m.–4 a.m., but its concentration decreased during the day.

In our study, we found that the highest concentration of melatonin at 24 h in the amphetamine dependent patients was significantly higher than opium dependent patients. In the opium dependent patients, melatonin levels at 16 h was higher than that of amphetamine dependents, that demonstrated disruption in the circadian cycle of melatonin secretion in these patients.

It is known that the prevalence of CRSD is high in substance abusers. There are evidences that links substance use to circadian rhythms disturbances, particularly whether circadian disturbance leads to substance abuse or substance dependency. Given the important role of circadian rhythms in regulating sleep, and that circadian disruption is often inherent to sleep disturbance, circadian mechanisms may account in part for sleep-substance abuse interactions (Hasler et al., 2012). However, in the present study, CRSD in both group were similar; however, the serum melatonin level is low and disrupted in both substance dependent groups. Taking this into consideration, although lowered melatonin levels might explain the susceptibility of CRSD, some investigations that involved the circadian system in regulating reward processing, indicating that circadian mechanisms may be directly linked to substance abuse independently of sleep pathways (Hasler et al., 2012; Honma and Honma, 2009; Webb et al., 2009). This finding is consistent with an animal study in which the circadian melatonin rhythm was shown to be entrained by the light-dark cycle, while the sleep-wake cycle free-ran in chronic treatment of methamphetamine in rats (Masubuchi et al., 2000).

On the other hand, in the study by Oyefeso et al., that revealed that opiate addicts more than controls report difficulty initiating sleep; difficulty maintaining sleep; inadequate sleep quality and quantity, the investigators disregarded circadian timing issues and demonstrated that sleeping at inappropriate times, which is associated with the nocturnal lifestyle of opiate addicts, disrupted nocturnal sleep and circadian rhythms. The investigators were implying that such measures were less likely to be a consequence of drug use directly (Oyefeso et al., 1997).

There have no study in which the effectiveness of melatonin treatment for sleep problems in substance abusers has been evaluated. Several studies have also indicated that melatonin is effective for the treatment of CRSD accompanied by neurodevelopmental disorders (Pillar et al., 2000; Miyamoto et al., 1999; Laakso et al., 2007; Gringras et al., 2012). In a randomized controlled study conducted by Laakso et al., the endogenous melatonin secretion profile in adult patients with neurodevelopmental disorders and predictive of the response to melatonin treatment for insomnia symptoms was investigated. They divided the subjects into two groups according to the 24-h patterns of serum melatonin, that is, higher (>16 pg/ml) and lower (≥16 pg/ml) melatonin level groups, and compared the effect of 1, 3, or 6 mg melatonin treatment between the two groups. Their results indicated that the low serum melatonin levels at night successfully predicted the desired treatment response to the administration of exogenous melatonin. Also, higher doses were not more effective than the lowest dose. (Laakso et al., 2007).

In the previous animal study by Permpoonputtana et al., the effect of d-amphetamine on hippocampal and prefrontal cortex neurons was investigated. In their study, after seven consecutive days injection of d-amphetamine for postnatal rats, they detected a downregulation of βIII-tubulin (a marker of premature neuron expression) and increase in glia fibrillary acidic protein (an astroglia marker) expression that implicated astrogliosis in both hippocampus and prefrontal cortex. Also, they found that melatonin pretreatment significantly attenuated the d-amphetamine-induced decrease in βIII-tubulin expression and prevented glial cell activation in both hippocampus and prefrontal cortex. They suggested that melatonin can attenuate the negative effect of d-amphetamine on neuronal cells and could be explored as a therapeutic treatment for d-amphetamine toxicity (Permpoonputtana et al., 2012).

Tocharus et al., in their physiological study showed that the induction of nitric oxide synthase (NOS) by amphetamine in microglial cells could be an important source of nitric oxide in CNS inflammatory disorders associated with the death of neurons. Pretreatment with melatonin significantly reduced NOS mRNA expression; so they suggested that melatonin be able to be used as a neuroprotective agent in amphetamine-induced toxicity (Tocharus et al., 2008).

Melatonin levels feed back to the melatonin sensitive neurons of the SCN (Pickard, 1994). So, production of melatonin in pineal gland is impaired which leads not only to disturbed sleep but also to dysregulation of circadian rhythmicity (Morin and Allen, 2006; Pickard, 1994).

Studies showed that substance abuse can affect SCN and alter circadian rhythm that enhances intake and motivations to drug seeking (Falcón and McClung, 2009; Kosobud et al., 2007). However, future studies need to determination the underlying mechanisms of the effects of substance abuse on SCN, circadian rhythm and melatonin production changes.

This study has several limitations. First, the sample size was small and, therefore, the application of the statistical analyses may be limited. Studies involving larger numbers of substance abuser patients are necessary to further clarify the relationship between CRSD and serum melatonin level in these patients. Second, randomized controlled trials will be necessary to confirm the effectiveness of exogenous melatonin administration for the treatment of sleep and circadian rhythm disturbance in substance abuser patients. Third, we did not use reliable objective measures such as polysomnographic or actigraphic findings, and reliance on sleep diaries recorded by the patients or family members could limit the accuracy of their nighttime disturbance. This may partly explain the lack of difference in some sleep parameters between the groups.

In conclusion, this study revealed a high prevalence of CRSD in substance dependent patients, possibly related to abnormal melatonin cycle. Similar prevalence of CRSD in both group and disruption in the circadian cycle of melatonin secretion in both groups demonstrated that circadian mechanisms may be directly linked to substance dependency independently of sleep pathways. Sleep and circadian rhythm-based interventions could play an important role in the prevention and treatment of substance use disorders.

## Conflict of interest

All authors declare that they have no conflicts of interest.

## Declaration of interest

The authors declare no conflict of interest.
